# The Role of Plant Growth-Promoting Bacteria in Soil Restoration: A Strategy to Promote Agricultural Sustainability

**DOI:** 10.3390/microorganisms13081799

**Published:** 2025-08-01

**Authors:** Mario Maciel-Rodríguez, Francisco David Moreno-Valencia, Miguel Plascencia-Espinosa

**Affiliations:** 1 Centro de Investigación en Biotecnología Aplicada (CIBA), Instituto Politécnico Nacional, Ex-Hacienda San Juan Molino, Carretera Estatal Tecuexcomac-Tepetitla Km 1.5, Tlaxcala 90700, Mexico; mmacielr1500@alumno.ipn.mx; 2 Secretaría de Ciencia, Humanidades, Tecnología e Innovación (SECIHTI)—Group “Ecology and Survival of Microorganisms”, Laboratorio de Ecología Molecular Microbiana, Centro de Investigaciones en Ciencias Microbiológicas, Instituto de Ciencias, Benemérita Universidad Autónoma de Puebla, Puebla 72570, Mexico

**Keywords:** abiotic stress, agricultural sustainability, Plant Growth Promoting Bacteria (PGPB), restoration of degraded soils, soil microbiota

## Abstract

Soil degradation resulting from intensive agricultural practices, the excessive use of agrochemicals, and climate-induced stresses has significantly impaired soil fertility, disrupted microbial diversity, and reduced crop productivity. Plant growth-promoting bacteria (PGPB) represent a sustainable biological approach to restoring degraded soils by modulating plant physiology and soil function through diverse molecular mechanisms. PGPB synthesizes indole-3-acetic acid (IAA) to stimulate root development and nutrient uptake and produce ACC deaminase, which lowers ethylene accumulation under stress, mitigating growth inhibition. They also enhance nutrient availability by releasing phosphate-solubilizing enzymes and siderophores that improve iron acquisition. In parallel, PGPB activates jasmonate and salicylate pathways, priming a systemic resistance to biotic and abiotic stress. Through quorum sensing, biofilm formation, and biosynthetic gene clusters encoding antibiotics, lipopeptides, and VOCs, PGPB strengthen rhizosphere colonization and suppress pathogens. These interactions contribute to microbial community recovery, an improved soil structure, and enhanced nutrient cycling. This review synthesizes current evidence on the molecular and physiological mechanisms by which PGPB enhance soil restoration in degraded agroecosystems, highlighting their role beyond biofertilization as key agents in ecological rehabilitation. It examines advances in nutrient mobilization, stress mitigation, and signaling pathways, based on the literature retrieved from major scientific databases, focusing on studies published in the last decade.

## 1. Soil Degradation Challenges Stemming from Excessive Agricultural Cultivation

Food security is a fundamental pillar of global health, human well-being, and economic stability [1]. Ensuring the availability, accessibility, and quality of food is essential to sustaining the world’s population [2]. However, while agriculture is indispensable for human survival, unsustainable and excessive practices can result in severe environmental consequences. Soil degradation driven by agricultural intensification, land-use changes, and poor soil management has emerged as a critical global threat, compromising ecosystem functions and food security worldwide [3]. One of the primary consequences of over-cultivation is nutrient depletion, which impairs soil fertility and long-term productivity [4,5]. This reduction in fertility diminishes the soil’s ability to sustain healthy crops and support balanced ecosystems [6,7]. The intensive use of fertilizers, often necessary to maintain high yields, exacerbates nutrient leaching, particularly of nitrogen and phosphorus, into deeper soil layers or groundwater, contributing to water contamination [8]. This process also promotes salt accumulation and soil acidification [9], further reducing soil quality and increasing reliance on external inputs, thereby perpetuating the cycle of degradation [10,11].

Intensive monoculture further aggravates this scenario by selectively depleting specific nutrients required by individual crop species, resulting in nutrient imbalances and declining yields over time [12,13,14,15,16]. This situation is compounded by excessive tillage [17] and the use of heavy machinery [18], which lead to soil compaction and the formation of a dense, impermeable layer known as the plow pan [19]. Compacted soils restrict root penetration and water infiltration, reducing porosity and limiting the availability of nutrients and oxygen, both essential for optimal plant growth [20]. Moreover, compacted soils are more susceptible to wind and water erosion, which removes fertile topsoil, degrades soil structure, and negatively impacts microbial biodiversity and ecosystem functionality [21,22].

Soil degradation also decreases crop resilience to disease and environmental stress [23] and promotes nutrient and organic matter runoff into nearby water bodies, contributing to pollution and eutrophication [24,25]. Excessive tillage and the use of disk plows disrupt soil aggregates, which are critical for maintaining soil porosity, thereby promoting the formation of compacted subsurface layers that may persist for years [26]. To mitigate these impacts, it is essential to implement sustainable soil management practices, such as crop diversification, the use of cover crops, and the incorporation of organic matter. These approaches improve soil structure, reduce compaction, and support soil health. Additionally, limiting machinery traffic through direct seeding techniques and designated access paths contributes to the long-term preservation of soil quality [27,28].

Soils host an extraordinary diversity of microorganisms, including bacteria, fungi, actinomycetes, and protozoa, which play critical roles in maintaining soil health and fertility [13,15]. However, excessive agricultural intensification has led to a decline in microbial biodiversity, with serious implications for soil function and the sustainability of agroecosystems [29]. The overuse of pesticides and herbicides significantly reduces populations of beneficial microbes such as nitrogen-fixing bacteria, mycorrhizal fungi, and actinomycetes, compromising essential ecosystem functions like organic matter decomposition, nutrient cycling, and plant health maintenance [30]. This reduction in microbial diversity negatively affects crop yields [31] and increases dependence on external inputs such as synthetic fertilizers and pesticides, with long-term economic and environmental repercussions [32].

**Figure 1:**
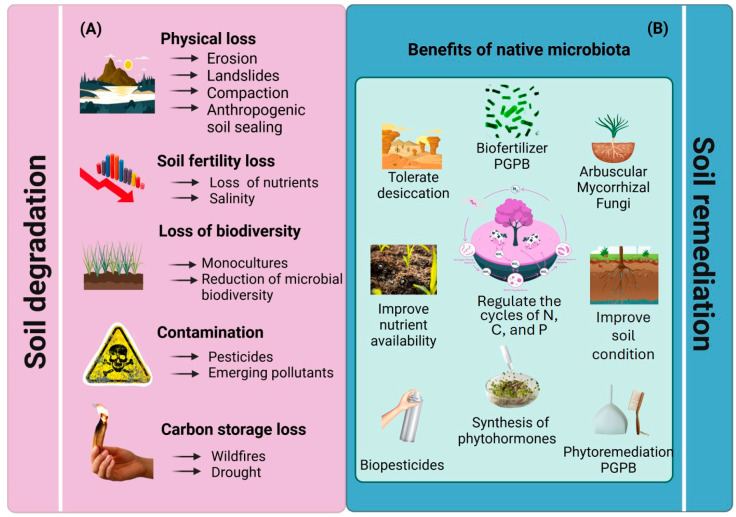
Factors contributing to soil degradation and microbiota-based remediation strategies. The primary causes of soil degradation (**A**) are predominantly anthropogenic, leading to adverse effects on soil structure and function, ultimately compromising its long-term sustainability. Microbiota-based remediation strategies (**B**) aim to restore soil integrity and functionality, promoting sustainable recovery. These strategies include the application of biofertilizers, biopesticides, and phytoremediation techniques.

The indiscriminate use of agrochemicals, combined with physical soil degradation, pollution, wildfires, and droughts, exerts direct pressure on soil microbial communities, significantly reducing their population density and diversity (Figure 1A). Additionally, pesticides and herbicides disrupt microbial ecosystems by selectively promoting the proliferation of certain taxa at the expense of native species, ultimately compromising soil functionality [33]. The widespread adoption of intensive monocultures further exacerbates this issue by creating selective environments that favor a narrow range of microbial species, thereby reducing overall diversity and weakening soil resilience to pathogens and environmental stressors [34]. These disturbances degrade natural habitats and disrupt critical biogeochemical cycles, limiting microbial activity, reducing soil porosity, and hindering the movement of air and water within the soil matrix [35].

To address these challenges, the adoption of sustainable agricultural practices is essential to restore soil structure, alleviate compaction, and promote microbial activity. Moreover, the application of PGPB plays a pivotal role in conserving and restoring soil microbial diversity. These bacteria constitute a diverse group of free-living or endophytic microorganisms that promote plant growth and development through a range of direct and indirect mechanisms. They contribute to soil health and resilience by facilitating phosphorus solubilization, nitrogen fixation, and the biosynthesis of plant growth regulators that regulate key physiological processes, including cell division, elongation, and differentiation [36] (Figure 1B). Furthermore, PGPB help mitigate abiotic stressors such as salinity and drought through the production of osmoprotectants and enzymes that alleviate ethylene-induced stress in plants [37,38]. In the context of drought, one critical limitation to the success of PGPB inoculants is desiccation, which reduces bacterial viability during formulation, storage, and post-application in dry soils. Therefore, the use of desiccation-tolerant strains is particularly advantageous, as they maintain cellular viability under conditions of low water availability and improve plant colonization efficiency [39]. This characteristic makes them highly suitable for application in arid and semi-arid regions [40].

This integrated approach fosters the sustainability of agricultural systems while preserving the long-term health of soils and the surrounding environment. By combining responsible agricultural practices with the targeted use of PGPB, soil fertility can be restored, crop productivity enhanced, and the reliance on synthetic chemical inputs reduced, ultimately contributing to a more sustainable and ecologically balanced agroecosystem [41,42].

## 2. Direct and Indirect Mechanisms of Plant Growth-Promoting Bacteria

**Figure 2:**
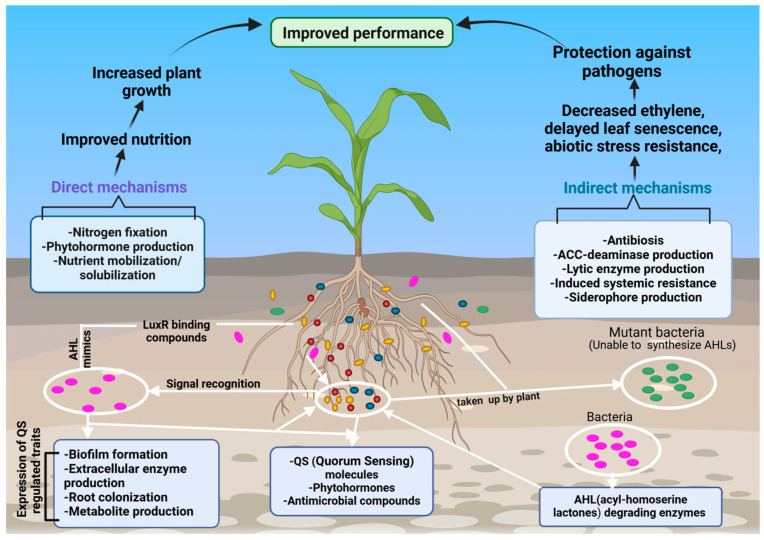
Direct and indirect mechanisms through which plant growth-promoting bacteria (PGPB) enhance plant performance via nutrient acquisition, stress mitigation, and pathogen suppression. Direct mechanisms include nitrogen fixation, phytohormone production, and nutrient solubilization, whereas indirect mechanisms involve ACC deaminase activity, antibiotic production, siderophore release, and induction of systemic resistance. Signal molecules, such as N-acyl-homoserine lactones (AHLs), mediate quorum sensing (QS), influencing processes like biofilm formation, enzyme secretion, and metabolite production. The plant microbiome includes different functional groups (colored ovals): AHL molecules involved in QS signaling (pink ovals); mutant bacteria unable to synthesize AHLs (green ovals); and other signaling compounds (e.g., LuxR-binding mimics and QS intermediates) that modulate plant–microbe communication (orange and blue ovals).

Plants have coevolved with a diverse microbiota that plays a pivotal role in supporting their growth, development, and health [43]. These microorganisms establish symbiotic associations with plant tissues, receiving carbon-rich compounds and other metabolites in exchange for beneficial services provided to the host [43]. This symbiosis dates to the early stages of terrestrial plant evolution and has enabled plants to overcome key challenges, including nutrient acquisition, abiotic stress tolerance, and protection against pathogens [44,45,46] (Figure 2).

In recent years, there has been an increasing interest in the use of microbial consortia, rather than single-strain inoculants, for restoring degraded soils. Numerous studies have demonstrated that PGPB consortia outperform individual strains due to functional complementarity among their constituent microorganisms. A recent meta-analysis revealed that microbial consortia inoculated into living soils exhibit superior plant growth-promoting effects compared to single-strain inoculants, primarily due to synergistic interactions involving mechanisms such as IAA production, phosphate solubilization, and stress tolerance induction [47]. Similarly, consortia comprising PGPB, arbuscular mycorrhizal fungi, and mineral-solubilizing microorganisms have been shown to significantly improve plant biomass and soil microbial activity in alluvially mined and degraded soils [48].

In saline and degraded environments such as Mediterranean Technosols, the application of bacterial consortia has yielded promising results. These consortia enhanced both germination and growth in lettuce (*Lactuca sativa*) while stabilizing the soil’s physicochemical properties, a key factor in fertility restoration [49]. Likewise, under drought conditions, the inoculation of maize (*Zea mays*) with bacterial consortia improved leaf water retention and facilitated a more efficient regulation of stress-related gene expression, compared with single-strain applications [50].

Collectively, these findings suggest that microbial consortia offer a more integrated and resilient approach to overcoming the limitations of degraded soils. The synergistic interactions among microbes enhance ecological stability, functional redundancy, and metabolic versatility, making consortia more effective and sustainable than single-strain inoculants [51].

Substantial progress has been made in characterizing the composition and dynamics of plant-associated microbial communities, as well as elucidating the functional attributes of key bacterial strains. PGPB have been isolated from a wide range of agriculturally and ecologically relevant plant species [52,53]. Building on these insights, research has focused on the bioactive compounds secreted by PGPB including phytohormones, secondary metabolites, antibiotics, and signaling molecules, which function as biostimulants and mediate plant responses to abiotic and biotic stress. In addition to these benefits, PGPB also promote plant health by enhancing nutrient availability and uptake, improving soil aggregation and modulating rhizosphere signaling. These combined effects improve overall plant development, stress resilience, and productivity. Nonetheless, while these microbial innovations show great promise, a deeper understanding of their molecular and ecological mechanisms remains essential for their effective, reproducible implementation in agricultural systems [54].

Given the potential of PGPB and the urgent challenges facing modern crop production, integrating microbial innovations into agronomic practices represents a critical strategy for enhancing agroecosystem sustainability. Understanding the underlying molecular and physiological mechanisms of PGPB is essential for effectively translating microbial innovations into practical strategies for soil restoration and sustainable agriculture. The following sections explore the primary mechanisms by which PGPB enhance plant growth, including biological nitrogen fixation, phosphate solubilization, siderophore production, and the synthesis of plant growth regulators [55].

### 2.1. Indirect Mechanisms

#### 2.1.1. Siderophores Production

Iron plays a fundamental role in plant photosynthesis, serving as an essential component of chlorophyll and participating in various biosynthetic pathways. However, the bioavailable iron fraction in soils is often insufficient to support optimal plant productivity [40]. To overcome this limitation, bacteria, fungi, and plants release specific low-molecular-weight compounds known as siderophores, which chelate iron from the surrounding environment [56].

Siderophores are primarily synthesized by bacteria to alleviate iron deficiency stress, thereby promoting plant growth. These microorganisms express surface receptors that regulate the uptake of ferric iron (Fe^3+^), ensuring its availability for metabolic processes [57]. Notably, siderophores produced by PGPB exhibit an exceptionally high affinity for Fe^3+^. Once the iron–siderophore complex is formed, it is recognized by specific receptors on the bacterial or plant cell surface, internalized, and subsequently reduced to ferrous iron (Fe^2+^) or released upon degradation of the siderophore, rendering iron bioavailable for cellular metabolism [56].

Importantly, siderophores produced by PGPB demonstrate significantly higher iron-binding affinities compared to those synthesized by plants or fungi, enabling PGPB to sequester substantial amounts of this micronutrient [58]. This trait enhances iron acquisition under limiting conditions and contributes to plant protection. By releasing siderophores with superior chelating capabilities, PGPB effectively outcompete pathogenic fungi and bacteria for iron, thereby restricting their growth and serving as an indirect biocontrol mechanism [59]. The efficacy of this antagonistic strategy is attributed to the fact that PGPB siderophores can outcompete fungal siderophores in iron chelation [60].

Structurally, microbial siderophores commonly possess functional groups such as hydroxamates and catecholates, in addition to carboxylates, citrates, or ethylenediamine moieties, which may coexist within the same molecule [61]. Hydroxamate-type siderophores are predominant in fungi, whereas catecholate-type siderophores, which exhibit stronger iron-binding capacities, are more characteristic of bacterial species [62]. In contrast, plant-derived siderophores, such as mugineic and avenic acids, belong to the amino carboxylic acid family and are distinguished by linear chains containing hydroxyl and amino functional groups that enhance metal chelation, including that of Fe^3+^. These compounds exhibit high chelation efficiency under certain conditions [63]. Additionally, bacterial siderophores can chelate other trivalent and divalent metal ions, although with significantly lower affinities than iron [64].

#### 2.1.2. Enzymatic Mechanisms in Indirect Plant Defense

Several microbial compounds contribute to indirect defense mechanisms, including hydrolytic enzymes produced by PGPB that exhibit antimicrobial activity and act as a barrier against pathogenic bacteria [65]. Among these, proteases play a significant role in plant protection by degrading pathogen-derived proteins. These enzymes, secreted by species such as *Bacillus clausii* and *Bacillus lentus*, are categorized based on their optimal pH into alkaline, acidic, and neutral proteases, each contributing to defense under specific soil conditions [66].

Another important mechanism involves catalase-positive bacteria that protect plant roots from oxidative damage caused by hydrogen peroxide (H_2_O_2_), thereby enhancing plant tolerance under oxidative stress. Notable catalase-producing strains include *Bacillus insolitus, Bacillus pasteurii, Bacillus laterosporus,* and *Staphylococcus aureus* [67,68].

Hydrogen cyanide (HCN), a volatile and highly toxic secondary metabolite, is also synthesized by certain rhizospheric bacteria. HCN interferes with cellular respiration and inhibits the growth of pathogenic fungi, nematodes, insects, and termites. Additionally, it acts as a natural herbicide by colonizing the rhizospheres of competing plant species and suppressing their growth without harming the host plant [68,69].

Amylases, another group of important enzymes, contribute to plant protection by degrading polysaccharides in the cell walls of phytopathogens. These enzymes are classified into α-amylases, β-amylases, and γ-amylases, with α-amylases being the most produced by endophytic bacteria associated with medicinal and crop plants [70]. Bacterial species from the genus *Bacillus*, such as *Bacillus licheniformis*, *Bacillus stearothermophilus*, and *Geobacillus bacterium*, are notable for their high amylolytic activity and are widely studied for their biocontrol potential [71].

Ureases also play a critical role in the rhizosphere by catalyzing the hydrolysis of urea into ammonium and carbon dioxide, producing ammonium (NH_4_^+^), a readily assimilable nitrogen source for plants [72]. Moreover, ureolytic bacteria contribute to biomineralization through calcite precipitation, which occurs via an increase in pH and carbonate ion production. This process is relevant not only for soil nutrient enhancement but also for environmental applications such as biocementation and the repair of microcracks in concrete [73].

### 2.2. Direct Mechanisms

#### 2.2.1. Plant Growth Regulators and Their Role in Plant Growth and Signaling

Plant growth regulators (PGRs) are essential chemical compounds that enable plants to adapt and respond to dynamic environmental conditions [74]. Both plants and microorganisms synthesize PGRs, including cytokinins and auxins, which influence a wide range of physiological and developmental processes. However, research on cytokinin biosynthesis remains limited due to their structural diversity and typically low endogenous concentrations, which complicate detection and quantification [75]. Unlike animal hormones, PGRs are not synthesized in specialized organs but can be produced in nearly all plant tissues. Currently, five major groups of PGRs are recognized as key regulators of plant development: auxins (AUXs), gibberellins (GBRs), cytokinins (CTKs), ethylene (ETH), and abscisic acid (ABA) [76].

In addition to these canonical PGRs, other signaling molecules with hormone-like activities play crucial roles in plant defense against herbivores and pathogens. These include salicylic acid (SA), nitric oxide (NO), jasmonic acid (JA), brassinosteroids (BRs), strigolactones (SLs), and systemin peptides [77]. Although nitric oxide is well recognized as a signaling molecule and metabolic intermediary, it is not yet classified as a PGR due to its inorganic nature [78]. Moreover, research on gibberellic acid (GA) biosynthesis, particularly its microbial pathways, remains relatively underexplored, with minimal progress reported in the past two decades [79]. Among all PGRs, IAA is the most extensively studied due to its central role in plant growth promotion, mediated by PGPB [80]. Both plants and microorganisms are capable of synthesizing IAA through various biosynthetic pathways, one of the most common being tryptophan (Trp)-dependent. Microbial IAA production is influenced by several physiological parameters, including temperature, pH, and the availability of carbon and nitrogen sources [81].

PGRs often undergo long-distance translocation within plants, a process that is essential for systemic signaling and inter-organ communication. This transport is facilitated through the phloem and xylem. For example, auxins synthesized in the apical buds are translocated basipetally to the roots via the phloem, while cytokinins produced in the roots are transported acropetally to the shoot meristems via the xylem. Additionally, certain PGRs, particularly weak organic acids, may passively diffuse across cellular membranes in their protonated form [82]. As research continues to advance, it is expected that new classes of plant signaling molecules will be identified, thereby enhancing our understanding of the complex regulatory networks governing plant growth and environmental adaptation.

#### 2.2.2. Contribution of Microbial Activity to Nutrient Solubilization and Plant Growth

Phosphorus (P) is a critical macronutrient for plant growth, and its deficiency significantly impairs physiological development and crop productivity [83]. Certain PGPB possess the enzymatic and metabolic capability to solubilize inorganic phosphorus compounds, such as tricalcium phosphate, hydroxyapatite, and rock phosphate, through the secretion of organic acids, primarily citric and gluconic acids. These acids chelate phosphate-associated cations via their hydroxyl and carboxyl functional groups, enhancing phosphorus bioavailability in the rhizosphere [84]. Recent research has highlighted the role of specific microbial enzymatic pathways, particularly those involving glucose dehydrogenase (gdh), the pyrroloquinoline quinone (pqq) gene cluster, and organic acid biosynthesis systems, in mineral phosphate solubilization [85]. For instance, the expression of the gdh gene, which encodes glucose dehydrogenase, has been positively correlated with enhanced phosphate solubilization in crops such as wheat and chickpea. Under tricalcium phosphate (TCP) stress, gdh expression showed up to a 1.59-fold increase, as quantified by qRT-PCR, compared with uninoculated controls [86]. Furthermore, the pqq gene cluster, particularly the pqqC gene, is critical for the biosynthesis of PQQ, a redox cofactor essential for GDH activity. This enzyme oxidizes glucose to gluconic acid, thereby acidifying the rhizosphere and mobilizing phosphate ions by displacing them from metal complexes. The presence and expression levels of pqqC have been closely linked to high phosphate-solubilizing efficiency in genera such as *Pseudomonas* and *Burkholderia*, positioning this gene as a potential molecular marker for selecting elite phosphate-solubilizing bacterial strains [87,88]. Meta-analytical evidence further supports the application of phosphate-solubilizing bacteria (PSB) as bio-inoculants, reporting increases in the available phosphorus in soil ranging from 8% to 73%, with the outcomes influenced by soil physicochemical characteristics and crop species [89,90,91].

Zinc (Zn) is another essential micronutrient, acting as a catalytic or structural cofactor in numerous enzymes involved in photosynthesis, hormone synthesis, and stress resistance. Specific PGPB can increase zinc bioavailability by releasing siderophores and organic acids that solubilize Zn from otherwise insoluble forms, such as ZnO and Zn_3_(PO_4_)_2_ [92,93]. For example, the strains of *Burkholderia cepacia* and *Acinetobacter baumannii* demonstrated zinc solubilization of up to 1.44 ppm under in vitro conditions. In pot trials using *Zea mays*, inoculation with these strains significantly enhanced shoot and root development, compared with non-inoculated controls. High-performance liquid chromatography (HPLC) analyses identified the presence of oxalic, maleic, tartaric, and fumaric acids in the rhizosphere, supporting their role in zinc mobilization and uptake [94].

Potassium (K), a major macronutrient, is essential for numerous plant processes including enzyme activation, osmoregulation, and stomatal function. Although potassium is naturally abundant in soils, a large proportion is sequestered in insoluble mineral forms, such as feldspar and mica, making it unavailable to plants [84]. Potassium-solubilizing bacteria (KSB), including *Bacillus aryabhattai* SK1-7 and *Pantoea vagans* ZHS-1, can release K^+^ ions through the secretion of organic acids such as citric, oxalic, and gluconic acids, which acidify the soil and chelate aluminum or silicon-based complexes [95,96]. *B*. *aryabhattai* SK1-7 was shown to solubilize potassium at a rate of 32.6%, releasing 10.8 μg/mL of K^+^ in vitro, while *P*. *vagans* ZHS-1 reached 20.3 mg/L under optimized fermentation conditions. Field trials have reported increases in soil-available potassium ranging from 2.7% to 40.5%, depending on mineral composition and environmental context [97]. Moreover, KSB play an important role in enhancing plant tolerance to abiotic stress by improving the cytosolic K^+^/Na^+^ ratio, which is critical for maintaining cellular homeostasis under saline conditions [98].

## 3. Physiological Mechanisms of Plant-Microorganism Interaction

The beneficial interaction between plants and bacteria occurs within a dynamic and complex environment, where molecular and biochemical signaling plays a central role in mediating communication between the two partners [99]. This symbiotic relationship is sustained by a continuous exchange of chemical signals that coordinate interactions between plants and free-living bacteria, resulting in mutual benefits for both organisms [100]. Among these microorganisms, PGPB enhance plant development through a combination of direct and indirect mechanisms, as previously described. This section focuses on the physiological mechanisms that underlie plant–microorganism interactions, with the objective of elucidating how PGPB exert their beneficial effects at the functional and physiological levels. Particular attention is given to signaling pathways, stress mitigation responses, and the modulation of plant metabolism triggered by microbial activity.

### 3.1. Bacterial Contribution to Plant Nutrient Acquisition

#### 3.1.1. Nitrogen

According to Liebig’s law of the minimum, nitrogen (N) is typically the most limiting nutrient for plant growth, followed by P [101]. N is an essential element for all living organisms, as it forms a fundamental component of nucleic acids and proteins [102]. Although atmospheric nitrogen (N_2_) is abundant, most organisms are unable to assimilate it directly. Only a specific group of bacteria and archaea, collectively known as diazotrophs, possess the enzymatic machinery to convert N_2_ into ammonia (NH_3_) via biological nitrogen fixation (BNF), a process catalyzed by the oxygen-sensitive nitrogenase enzyme complex [103].

Diazotrophic bacteria do not freely excrete ammonia, as nitrogen fixation and assimilation are tightly coupled and regulated through complex molecular pathways [104]. These include transcriptional regulators, post-translational protein-modifying enzymes, and PII signal transduction proteins that modulate the expression of nitrogen metabolism genes [105,106,107]. In diazotrophic proteobacteria, these pathways interact with the nitrogen-fixation regulator NifA, ensuring nitrogenase expression is tightly regulated according to nitrogen demand [108]. The ammonia produced during BNF diffuses across the bacterial membrane but is promptly recovered by the ammonium transporter AmtB, despite the high energetic cost of the process [109]. Notably, BNF requires approximately 16 moles of ATP per mole of fixed N_2_, underscoring its energy-intensive nature [110].

Nitrogenase is a highly conserved metalloenzyme complex encoded by the nif gene cluster and plays a central role in nitrogen fixation [111]. In addition to the nif genes, other key functional genes, such as *cbbL*, *nifH*, *amoA*, and *apsA*, are commonly used to assess microbial contributions to nitrogen and sulfur cycling in soil ecosystems [112]. The nitrogenase complex comprises two metalloproteins: molybdenum–iron nitrogenase (MoFe, encoded by nifDK) and iron protein nitrogenase reductase (Fe, encoded by nifH) [113]. The Fe protein transfers electrons to the MoFe protein via MgATP hydrolysis, enabling N_2_ reduction at the MoFe_7_S_9_-homocitrate cofactor active site [114,115].

The fully functional nitrogenase enzyme is composed of a γ_2_ homodimer (NifH) and an α_2_β_2_ heterotetramer (NifD/NifK), where the MoFe_7_S_9_ cluster located within the α subunit (NifD) is responsible for catalyzing N_2_ reduction [116]. In some microorganisms, such as *Azotobacter vinelandii* and *Methanosarcina acetivorans*, the molybdenum cofactor can be substituted by iron (Anf) or vanadium (Vnf), forming alternative nitrogenase variants. Among these, FeMo nitrogenase is the most efficient, followed by Anf, and Vnf [117]. Due to the oxygen sensitivity of nitrogenase, diazotrophs have evolved protective adaptations, including the development of specialized structures or oxygen diffusion barriers, to maintain microaerobic conditions during nitrogen fixation [118].

**Figure 3:**
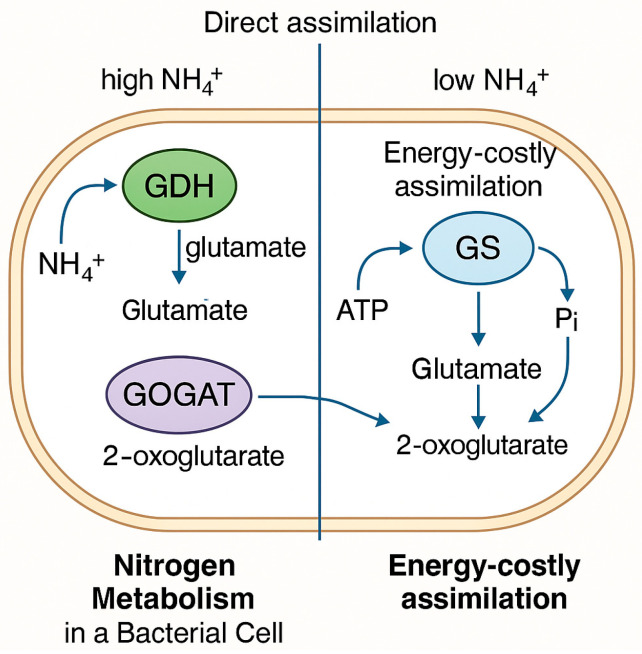
Molecular pathways of ammonium assimilation in bacteria. Ammonium (NH_4_^+^) is assimilated into glutamate via two main enzymatic pathways. The GDH (glutamate dehydrogenase) pathway catalyzes the reductive amination of 2-oxoglutarate to produce glutamate using NADPH. This route predominates under high ammonium availability and low energy/carbon conditions. Alternatively, the GS-GOGAT (glutamine synthetase–glutamate synthase) pathway is the primary mechanism under low ammonium concentrations. It involves the ATP-dependent conversion of glutamate into glutamine by GS, followed by the transfer of the amide group from glutamine to 2-oxoglutarate by GOGAT, forming two molecules of glutamate. This pathway provides a higher affinity for ammonium and is more energy-intensive, contributing significantly to nitrogen assimilation under nutrient-limited conditions.

Once ammonia is produced, bacteria assimilate it primarily via two metabolic pathways: the glutamine synthetase–glutamate synthase (GS-GOGAT) pathway and the glutamate dehydrogenase (GDH) pathway. The GDH pathway operates under high ammonium and low carbon/energy availability as it is ATP-independent and characterized by a high Km (~1 mM) for ammonium [119]. In contrast, the GS-GOGAT pathway predominates under low ammonium and high energy/carbon conditions. This pathway involves the ATP-dependent conversion of glutamate into glutamine by glutamine synthetase (GS), followed by the NADPH-dependent transfer of the amide group from glutamine to 2-oxoglutarate by glutamate synthase (GOGAT), producing two molecules of glutamate [120] (Figure 3). This GS-GOGAT pathway represents the primary route for ammonia assimilation in most bacteria [121].

Glutamate plays a pivotal role as a nitrogen carrier in plants, facilitating the translocation of nitrogen from the roots to aerial tissues via the xylem [122]. Once transported, glutamate-derived amino acids are efficiently utilized in key physiological processes, including seed germination, protein and nucleic acid biosynthesis, and the synthesis of other essential metabolites required for plant growth and development [123]. Furthermore, glutamate-derived intermediates modulate critical metabolic pathways such as photosynthesis, respiration, and stress-response signaling, thereby ensuring optimal physiological function and enabling plants to adapt to fluctuating environmental conditions [124]. The central role of glutamate in integrating nitrogen into a wide array of biomolecules underscores the complexity and efficiency of plant nitrogen metabolism, supporting sustained growth and productivity.

#### 3.1.2. Phosphorus

After nitrogen, phosphorus (P) is the second most limiting essential mineral nutrient for plant growth, as it is only absorbed in its soluble monobasic (H_2_PO_4_^−^) or dibasic (HPO_4_^2−^) forms [125]. In soils, P is predominantly found in inorganic forms, either adsorbed onto soil mineral surfaces or in poorly available precipitates. It is also present in organic forms, where it is incorporated into biomass or associated with soil organic matter [100].

P is one of the nineteen essential elements for plant life and plays a central role in energy capture, storage, and transfer. It is a key structural component of DNA, RNA, and phospholipids in both plant and animal cells [83]. It participates in fundamental physiological processes including photosynthesis, root development, stem elongation, flower and seed formation, crop maturation, energy metabolism, cell division and expansion, nitrogen fixation in legumes, disease resistance, starch biosynthesis, and genetic information transfer [126]. Moreover, adequate phosphorus availability is critical for the formation of reproductive primordia during early plant development [127]. Thus, phosphorus is indispensable for nearly all aspects of plant physiology.

Within the soil–plant system, approximately 90% of the total phosphorus is present in the soil matrix, while less than 10% is found in the soil solution. However, most soil phosphorus is bound to particles or minerals such as apatite, hydroxyapatite, and oxyapatite, making it largely unavailable to plants. Organic phosphorus compounds, such as inositol phosphates (phytate), phosphomonoesters, and phosphotriesters, must undergo mineralization to become bioavailable [128].

Phosphate-solubilizing bacteria (PSB) are key members of the plant microbiome that enhance the conversion of insoluble organic and inorganic phosphate forms into bioavailable phosphorus, particularly under phosphorus-deficient conditions [129]. This microbial activity is essential for improving phosphorus availability and supporting plant growth in nutrient-depleted soils [103].

The primary mechanism employed by PSB involves acidification of the surrounding soil environment through the secretion of organic acids or proton release, thereby increasing phosphorus solubility. In alkaline soils, phosphorus commonly precipitates as calcium phosphates such as fluorapatite or francolite, which are poorly soluble [130]. As soil pH rises, the predominant phosphate species shift toward divalent (HPO_4_^2−^) and trivalent (H_2_PO_4_^−^) forms. PSB metabolize carbon sources, such as glucose, to release organic acids as metabolic byproducts via oxidative respiration or fermentation [131]. The type and concentration of these acids, particularly di- and tricarboxylic acids, strongly influence the efficiency of phosphate solubilization. Common organic acids involved include gluconic, lactic, citric, isovaleric, succinic, glycolic, oxalic, formic, 2-ketogluconic, and acetic acids [132].

Among these, gluconic acid and 2-ketogluconic acid are the most frequently reported in mineral phosphate solubilization [133]. Gluconic acid is predominantly produced by *Pseudomonas* spp. [134,135] and *Burkholderia* spp. [136,137], while 2-ketogluconic acid is synthesized by *Nguyenibacter* sp. [138] and *Rhizobium tropici* [139]. *Priestia megaterium* and *Bacillus velezensis* have been reported to produce lactic, acetic, and citric acids, whereas *Enterobacter* spp. mainly produces acetic acid [140]. Gram-negative bacteria tend to be more efficient at solubilizing mineral phosphates due to their broader capacity to excrete organic acids into the rhizosphere [141].

Mineralization also plays a vital role in P cycling by converting complex organic P compounds—such as sugar phosphates, nucleic acids, phospholipids, phytic acid, phosphonates, and polyphosphates—into inorganic phosphate (Pi) [142]. The mineralization and immobilization of organic P are critical processes in the biogeochemical cycling of this nutrient, particularly in agricultural and degraded soils. Through these mechanisms, PSB increase P availability to plants, contributing to improved soil fertility and crop productivity [143].

PSB facilitate the mineralization of organic P in soils by producing phosphatase enzymes that hydrolyze organic phosphate compounds, thereby releasing Pi in a bioavailable form for plant uptake. Phosphatase activity has been reported in both Gram-positive bacteria, such as *Bacillus* spp. [130], and Gram-negative bacteria, including *Burkholderia* sp., *Gluconacetobacter* sp. [136], and *Pseudomonas* sp. Additionally, filamentous fungi from the genera *Aspergillus* and *Penicillium* are known producers of phosphatases, further contributing to phosphorus cycling in soil ecosystems [144].

Phytases, a subclass of phosphatases, exhibit variations in their cellular localization between bacterial groups. In Gram-negative bacteria, phytases are typically located intracellularly or within the periplasmic space [145], while in Gram-positive bacteria, these enzymes are predominantly secreted extracellularly. Two main classes of phytases have been identified: acidic and alkaline phytases [146]. Acidic phytases, which display optimal activity at pH 2.5–5.5, belong to the histidine acid phosphatase (HAP) family and are characterized by a conserved RHGXRXP motif near the N-terminal region [147]. These enzymes generally function without the need for cofactors and are frequently synthesized by Gram-negative bacteria and fungi. In contrast, alkaline phytases, which operate optimally at pH 6–8, constitute a distinct enzymatic class. They are notable for their high substrate specificity toward phytic acid, and their requirement for calcium ions to maintain catalytic activity [148].

Another important mechanism by which PSB enhance phosphorus availability is through chelation. This process involves the production of organic and inorganic acids that solubilize mineral-bound phosphate by chelating metal cations and competing with phosphate for sorption sites on soil particles [142]. Chelating acids stabilize insoluble metal–phosphate complexes, particularly those involving aluminum and iron oxides, thereby releasing phosphate into the soil solution [149]. Functional groups such as hydroxyl and carboxyl moieties in these acids form stable complexes with the metal cations associated with phosphate, facilitating its conversion into plant-available forms [150]. In addition to organic acids, PSB can also produce inorganic acids such as carbonic acid, hydrogen sulfide, nitric acid, and hydrochloric acid that contribute to phosphate solubilization through similar mechanisms [151].

### 3.2. Bacterial Modulation of Plant Growth Regulator Pathways and Signaling Mechanisms

#### 3.2.1. Plant Growth Regulators: How Do PGPB Influence Growth Regulator Signaling Networks to Enhance Growth and Stress Tolerance?

PGRs are pivotal for plant growth, development, and stress adaptation, acting as central nodes in signaling networks. This section explores how PGPB synthesize or modulate phytohormones including auxins, cytokinins, ABA, ethylene, and gibberellins under normal and stress conditions. These microbial contributions optimize root and shoot architecture, improve nutrient acquisition, and activate defense responses, thus reinforcing plant resilience under biotic and abiotic challenges.

PGRs regulate plant morphology by stimulating both root and aerial systems, facilitating resource acquisition and stress resilience [152]. Beyond growth promotion, they act as molecular signals that mediate responses to biotic and abiotic stressors [153]. Numerous rhizospheric bacteria have been reported to produce PGRs that improve nutrient acquisition and modulate endogenous growth regulator balances, thereby supporting plant vigor and stress resilience [154].

Auxins (IAA): How does microbial IAA shape root system architecture and stress responses?

IAA is the most widely studied and prevalent natural auxin synthesized by PGPB through Trp-dependent pathways, particularly the indole-3-pyruvic acid (IPyA) route [155]. Microbial IAA regulates root elongation, lateral root initiation, and vascular differentiation, enhancing soil exploration and nutrient acquisition [156]. IAA also functions as a signaling molecule that modulates gene expressions associated with plant stress responses, including drought, salinity, nutrient limitation, and temperature extremes [157].

At the cellular level, IAA stimulates cell expansion by enhancing protein synthesis and loosening cell walls, which collectively contribute to improved root system architecture [158]. The accumulation of auxin in the rhizosphere is often triggered by endogenous signaling cues or organogenesis driven by environmental stimuli that activate key regulatory genes involved in cell division and tissue differentiation [159,160].

At the molecular level, auxin signaling begins with the perception of IAA by TIR1/AFB receptor proteins, components of the SCF (Skp1-Cullin-F-box) E3 ubiquitin ligase complex [161]. This interaction triggers the ubiquitination and degradation of Aux/IAA repressors, releasing auxin response factors (ARFs) to regulate the transcription of auxin-responsive genes [162]. These ARFs bind to auxin response elements (AuxREs) in the promoter regions of the target genes, working in concert with other transcriptional regulators to fine-tune gene expression in response to developmental and environmental signals [163]. These pathways coordinate developmental processes and stress adaptation by activating gene families such as GH3, which modulates IAA homeostasis, and SAUR (Small Auxin Upregulated RNA), which promotes cell expansion under environmental challenges [164,165,166,167].

Cytokinins: How do PGPB-derived cytokinins sustain meristem activity and shoot growth?

Cytokinins are purine-derived regulators that promote cell division and sustain meristematic activity, which is essential for developmental plasticity and stress adaptation [168,169,170,171,172]. By maintaining totipotent stem cell populations in apical meristems, they coordinate organogenesis and influence plant architecture [75]. Beyond growth regulation, cytokinins integrate environmental cues into developmental programs, supporting adaptive responses under abiotic stress.

Gibberellins: How Do Microbial Gibberellins Support Stem Elongation and Seed Germination?

Gibberellins (GAs) represent the most structurally diverse group of plant growth regulators, comprising over 100 diterpenoid molecules with distinct bioactivities [173]. These compounds are pivotal for developmental transitions, particularly stem elongation and seed germination, and act through signaling networks that integrate growth cues with environmental conditions [174]. While plant biosynthesis pathways are well characterized, microbial production remains less explored; however, evidence indicates that symbiotic bacteria, such as rhizobia, synthesize GAs alongside auxins and cytokinins during early nodulation and active cell division, suggesting a role in modulating host development under specific physiological contexts [175].

Abscisic Acid (ABA): How Does ABA Signaling Mediate Stress Responses and Water Balance?

Abscisic acid (ABA) functions as a central regulator of plant responses to drought and osmotic stress by modulating stomatal closure and growth adjustment [176,177]. Beyond its intrinsic signaling role, ABA dynamics are influenced by rhizospheric microorganisms. PGPB indirectly regulate ABA homeostasis through mechanisms affecting its synthesis, degradation, and transport, thereby enhancing stress adaptation. These effects interact with plant-driven processes such as apoplastic pH shifts, β-glucosidase activity, and vascular redistribution, collectively fine-tuning water balance and stress signaling under adverse conditions [178].

Ethylene: How Do PGPB Reduce Ethylene-Induced Growth Inhibition Under Stress?

**Figure 4:**
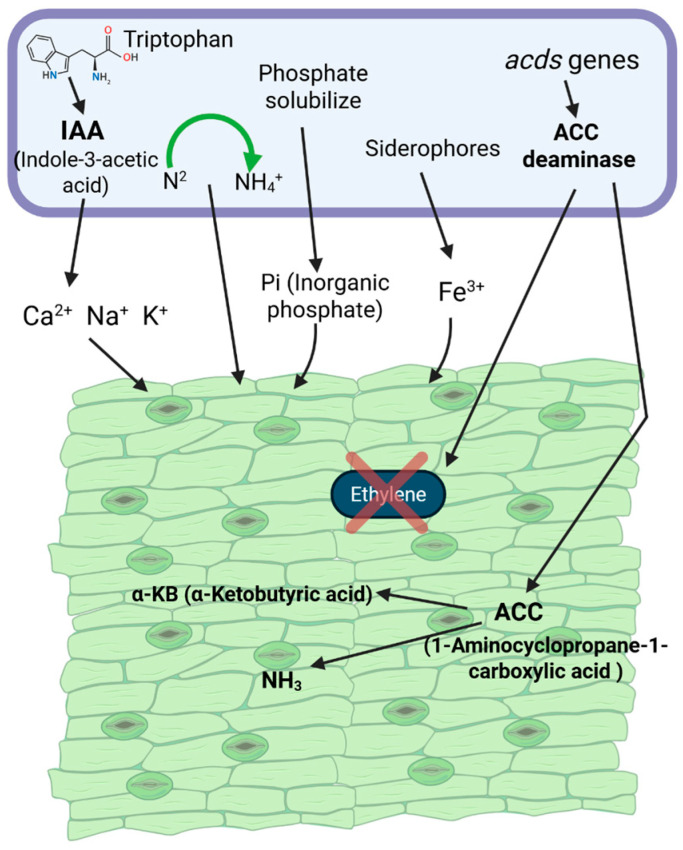
Mechanisms by which the PGPB enhance plant growth and mitigate stress. PGPB synthesize indole-3-acetic acid (IAA) from tryptophan, stimulating root elongation and lateral root development. They contribute to nitrogen (N_2_) fixation, converting atmospheric N_2_ into ammonium (NH_4_^+^), and solubilize inorganic phosphate (Pi) via the production of organic acids, increasing phosphorus bioavailability. Siderophore production by PGPB facilitates iron (Fe^3+^) acquisition, improving micronutrient uptake. Additionally, PGPB express the acdS gene encoding ACC deaminase, which degrades 1-aminocyclopropane-1-carboxylic acid (ACC)—the direct precursor of ethylene—into α-ketobutyrate (α-KB) and ammonia (NH_3_), thereby lowering ethylene levels and alleviating its inhibitory effects on plant growth under stress. PGPB also modulate ion uptake (Ca^2+^, Na^+^, and K^+^), promoting ionic balance and osmotic adjustment. Collectively, these mechanisms enhance plant nutrient acquisition, support root system development, and improve tolerance to abiotic stressors such as salinity and nutrient deficiency.

Ethylene is a key signaling molecule that mediates plant responses to biotic and abiotic stress, but excessive accumulation under stress inhibits root elongation and overall growth [179,180,181,182,183]. PGPB alleviate this effect primarily through the activity of ACC deaminase, encoded by the *1-aminocyclopropane-1-carboxylate deaminase gene (acdS)*, which hydrolyzes 1-aminocyclopropane-1-carboxylic acid (ACC), the immediate precursor of ethylene, into ammonia and α-ketobutyrate [184]. This reaction lowers ethylene biosynthesis and mitigates its inhibitory impact on root architecture (Figure 4) [185]. ACC exuded by roots is readily metabolized by rhizospheric bacteria, creating a buffering system that enhances stress resilience. By regulating ethylene levels, PGPB support root development, nutrient uptake, and plant adaptation under adverse conditions, establishing a critical mechanism for sustainable crop productivity in degraded soils [186].

#### 3.2.2. Volatile Organic Compounds (VOCs): How Do Bacterial VOCs Activate Defense Pathways and Improve Stress Resilience?

Volatile organic compounds (VOCs) act as airborne chemical mediators enabling plant–microbe and microbe–microbe communications. This subsection explores how PGPB-derived VOCs enhance plant defenses and physiological stability.

In addition to synthesizing PGRs, PGPB produce VOCs that play pivotal roles in long-distance chemical signaling within microbial communities and between microorganisms and plants in the rhizosphere [155]. These low-molecular-weight, lipophilic molecules are generated through diverse metabolic pathways and act as diffusible chemical messengers that mediate interkingdom communication [174]. A broad spectrum of VOCs such as ketones, terpenoids, alcohols, alkanes, alkenes, and sulfur-containing compounds is produced by both bacteria and fungi [187]. Among these, compounds like 2-heptanol, 2-undecanone, and pentadecane have been recognized as key signals involved in shaping root system architecture and promoting root–PGPB interactions [188,189]. Upon root detection of VOCs, a significant modulation of root development occurs, enhancing colonization and establishing a favorable rhizospheric environment for plant growth.

VOCs fulfill two major functional roles: (i) as semiochemicals facilitating inter- and intra-organismal signaling, and (ii) as antimicrobial agents that suppress pathogenic microorganisms. Notable VOCs with dual roles include pyrrolnitrin (PRN) and 2,4-diacetylphloroglucinol (DAPG) produced by species of *Pseudomonas*, *Butiauxella*, and *Serratia,* which contribute to plant protection through both direct pathogen inhibition and the activation of plant immune responses [190,191,192,193]. These compounds modulate hormonal signaling networks, such as the jasmonate and salicylate pathways, leading to the activation of defensive gene expression, accumulation of antimicrobial compounds, and reinforcement of structural barriers [194,195]. This process, referred to as induced systemic resistance (ISR), primes the plant to mount accelerated and robust responses upon subsequent pathogen encounters [196].

In addition, VOCs influence quorum sensing (QS) by disrupting intra- and interspecies bacterial communication systems [197], thereby altering population-level behaviors such as biofilm formation and secondary metabolite production. VOCs have also been shown to regulate genes involved in hormone signaling, particularly auxin pathways impacting root morphology and enhancing defenses against herbivorous insects [198]. Furthermore, the contribution of VOCs extends to enhancing plant tolerance to abiotic stressors (e.g., drought, salinity) and modulating microbial traits such as virulence and biofilm architecture [199,200,201,202]. Importantly, the volatile profile produced by PGPB is highly genotype- and species-specific, reflecting their metabolic diversity and ecological roles in shaping rhizosphere interactions and community dynamics [203]. These VOC-mediated mechanisms exemplify the sophisticated biochemical strategies employed by PGPB to support plant fitness, resilience, and ecosystem functionality.

#### 3.2.3. Quorum Sensing Detection

**Figure 5:**
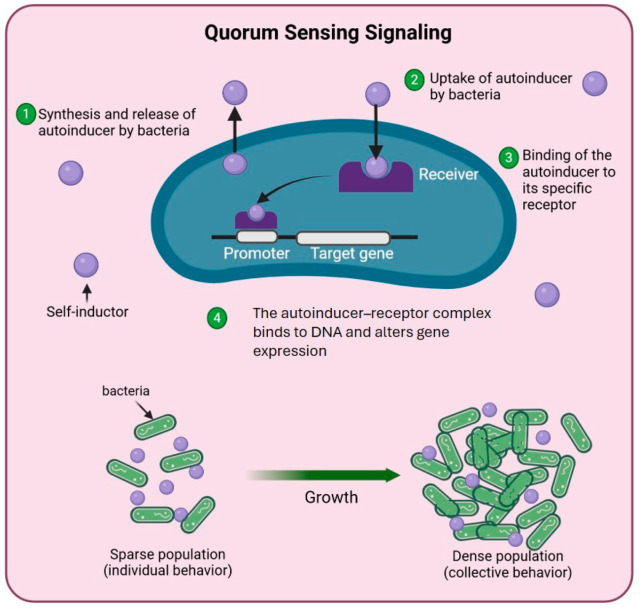
In low-density populations, bacteria exhibit individual behaviors, whereas in high-density populations, they transition to collective behavior through the production, release, and detection of signaling molecules known as autoinducers. This QS mechanism regulates gene expressions associated with group behaviors, including biofilm formation, virulence, and extracellular enzyme production.

Inter- and intraspecies communication in the rhizosphere is primarily mediated through QS signaling molecules, which enable microbial communities to synchronize and coordinate collective behaviors [204]. This cell-to-cell communication system is essential for the successful colonization of plant roots by PGPB and the modulation of microbial community dynamics [205]. QS signals, also referred to as autoinducers, allow bacteria to sense population density and regulate gene expression accordingly. These signals operate at both the intraspecific (within the same species) and interspecific (between different species) levels, influencing diverse microbial phenotypes such as conjugation, virulence, and rhizospheric competition as well as the biosynthesis of hydrolytic enzymes and secondary metabolites [102,155] (Figure 5).

**Figure 6:**
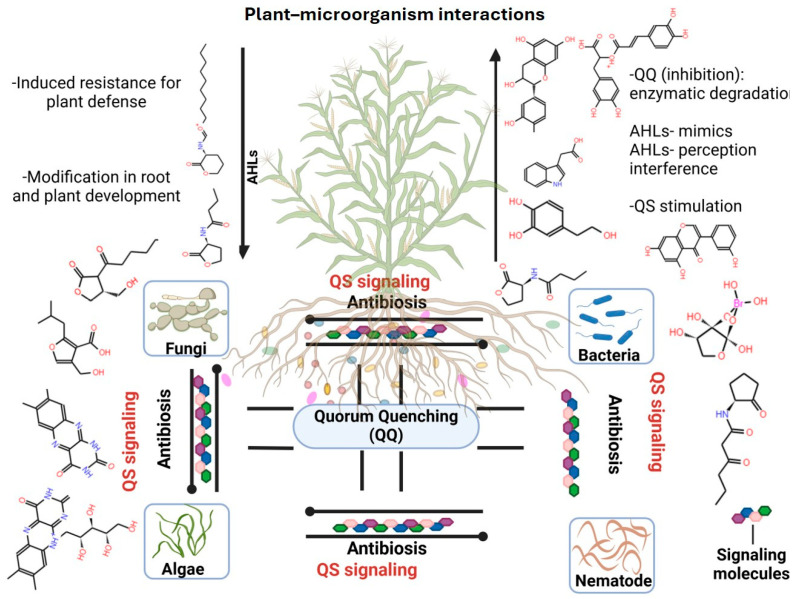
Plant–microorganism interactions mediated by signaling molecules and communication mechanisms. Plants release chemical compounds that influence microbial behavior in the rhizosphere through processes such as quorum sensing (QS) and antibiosis. In response, microorganisms produce specific signaling molecules, including AHLs and other chemical signals, which can induce plant resistance, modulate root development, and shape the surrounding microbiota. Additionally, quorum quenching (QQ) serves as a regulatory mechanism that degrades or interferes with QS signals, modulating inter-organism interactions and controlling processes such as virulence and microbial competition. Colored bars and spheres in the figure represent distinct signaling pathways and microbial interactions associated with QS, antibiosis, and QQ processes. Color coding is used to visually differentiate interactions and does not correspond to specific compounds or taxa.

In Gram-negative bacteria, the predominant class of QS molecules are N-acyl homoserine lactones (AHLs) [206]. These small, diffusible molecules are synthesized by LuxI-family enzymes and detected by LuxR-type receptor proteins, triggering transcriptional changes that orchestrate a wide array of physiological responses. AHL-mediated QS regulates crucial processes including plant–microbe symbioses, root morphogenesis, stress tolerance, the activation of induced resistance pathways, nutrient assimilation, and the maintenance of hormonal homeostasis in plants [207] (Figure 6). Numerous rhizospheric bacteria, such as species of *Serratia*, *Aeromonas*, and *Acinetobacter* as well as various proteobacteria, have been documented to both produce and perceive AHL signals [208,209]. This capability enables them to coordinate group behaviors that enhance rhizosphere colonization, biofilm formation, and plant growth promotion. The prevalence of AHL-mediated QS in the rhizosphere underscores its critical role in shaping microbial assemblages, facilitating cooperative and competitive interactions, and optimizing plant–microbe symbiotic efficiency. Ultimately, QS contributes to the establishment of a resilient, dynamic, and functionally integrated rhizospheric ecosystem that supports sustainable plant productivity.

Some Gram-negative bacteria, such as *Burkholderia* spp., produce a unique class of quorum sensing (QS) molecules known as diffusible signaling factors (DSFs). Chemically defined as cis-11-methyl-2-dodecenoic acid, DSFs facilitate population-level coordination and enhance innate immune responses in several crops, thereby strengthening their resistance to pathogen attacks [197,210].

In addition to canonical autoinducers like AHLs and DSFs, antibiotics at subinhibitory concentrations have also been recognized as signaling molecules within QS networks [211]. Rather than exerting bactericidal effects, these compounds function as intraspecific and interspecific chemical cues that regulate gene expression, mediate stress responses, and modulate behaviors such as biofilm formation, secondary metabolite production, and sporulation [195]. This dual functionality of antibiotics as both antimicrobial agents and signaling molecules underscores their ecological relevance in bacterial communication and community structuring [212].

Moreover, fungal species contribute to QS-mediated cross-kingdom interactions by producing a wide range of signaling molecules, including γ-heptalactone, γ-butyrolactone, tyrosol, farnesol, and dodecanol, many of which enable communication with bacterial partners or competitors [213]. Notably, *Saccharomyces cerevisiae* secretes alcohol-based signaling molecules associated with morphogenesis and stress adaptation during specific developmental stages [214]. These fungal–bacterial QS-mediated interactions represent sophisticated evolutionary adaptations that enable microbes to optimize colonization, outcompete rivals, and modulate host responses. Such communication systems are particularly important in the rhizosphere, where space and resources are limited [215]. Through these cross-kingdom signaling networks, microorganisms coordinate their physiological activities, influence the microbial community structure, and adaptively respond to fluctuating environmental conditions. This dynamic interplay of cooperation and competition reinforces the ecological fitness of both fungi and bacteria and contributes to niche specialization and functional resilience within complex soil ecosystems [216].

### 3.3. Plant Molecular Signaling and Microbial Communication in the Rhizosphere

In addition to the signaling molecules synthesized by PGPB, plants actively release a diverse array of compounds through root exudation. These exudates encompass low- and high-molecular-weight substances, including volatile and non-volatile organic compounds, which play a central role in mediating plant–microbe interactions in the rhizosphere [217]. By shaping the composition and activity of the surrounding microbiota, these exudates foster beneficial associations that enhance plant health, nutrient uptake, and stress resilience [218].

**Figure 7:**
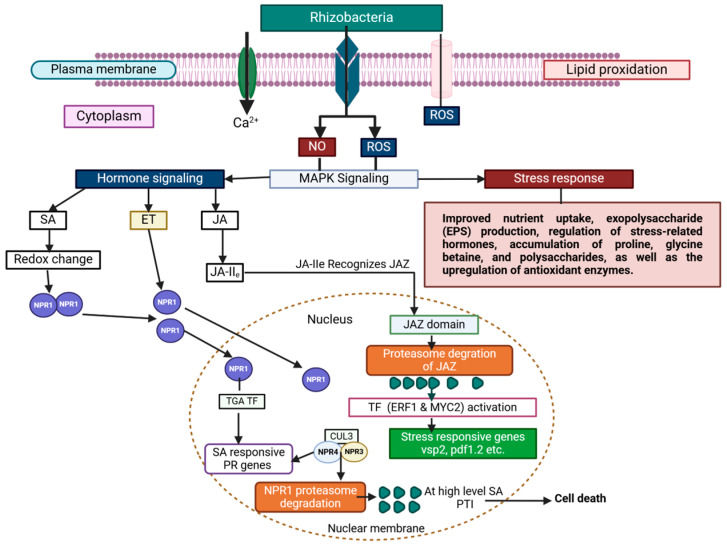
Signaling pathways involved in plant stress responses induced by pathogens and rhizobacteria. External signals, including reactive oxygen species (ROS), nitric oxide (NO), and calcium (Ca^2+^) fluctuations, activate hormonal signaling pathways and MAPKs, which regulate gene expression to enhance defense mechanisms and stress adaptation.

Plants perceive and respond to environmental cues through the activation of multiple signaling networks, notably mitogen-activated protein kinase (MAPK) cascades, which are integral to both abiotic and biotic stress responses. These cascades are tightly modulated by PGRs, which orchestrate defensive and developmental processes [219,220,221]. Through the integration of endogenous and exogenous signals, plants fine-tune their physiological responses to environmental stimuli, enabling survival and optimal growth. MAPK cascades are highly conserved across plant taxa and are composed of three sequentially acting kinases: MAPKKK, MAPKK, and MAPK, which transmit signals through phosphorylation events [222]. Although each cascade is typically activated by specific stimuli, crosstalk among pathways allows for nuanced coordination and amplification of cellular responses. Furthermore, MAPKs interact with other signaling mediators to generate highly specific outputs, contributing to developmental plasticity and enhanced defense signaling [223] (Figure 7). These cascades are implicated in the regulation of growth, root architecture, hormone signaling, and responses to drought, salinity, and pathogen invasion [224]. The versatility and modularity of MAPK signaling provide plants with a sophisticated regulatory framework to adapt to rapidly changing environmental conditions while optimizing microbial recruitment and symbiosis in the rhizosphere [225].

The metabolites secreted by both plants and PGPB act as chemical messengers that shape the structure and function of rhizospheric microbial communities. These specialized compounds, including flavonoids, terpenes, amino acids, sugars, and organic acids, play a dual role in microbial recruitment and signaling modulation, thereby enhancing plant–microbe compatibility [226]. In turn, microbial communities influence plant metabolic responses, forming a feedback loop of dynamic chemical communication.

This cross-kingdom signaling network is essential for the structuring and functional adaptation of the rhizomicrobiome. The diverse chemical cues released by plants selectively promote beneficial taxa and suppress antagonistic ones, resulting in microbiome assemblies that confer ecological advantages to the host. Moreover, microbial and plant-derived signaling molecules can be harnessed to modulate plant physiology and resilience through biotechnological interventions [227]. Overall, these molecular interactions govern key biological processes including plant development, gene regulation, hormonal homeostasis, and immunity, thus ensuring a mutually beneficial and finely balanced relationship between plants and their associated microbiota [155].

## 4. PGPB-Mediated Soil Restoration of Soils Degraded by Excessive Cultivation

**Figure 8:**
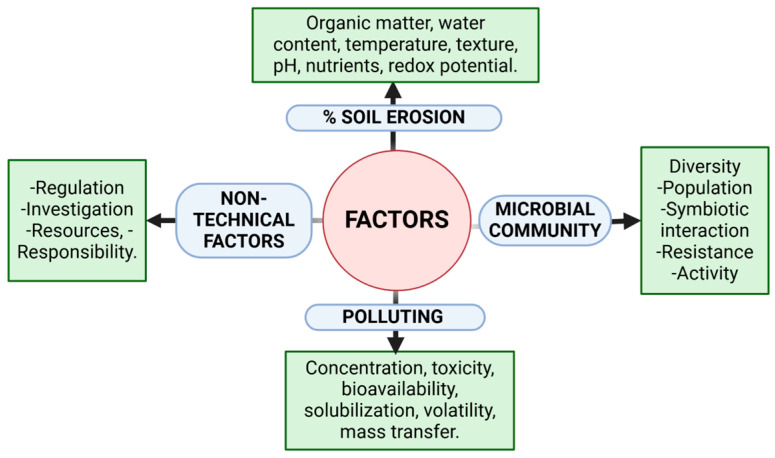
Key factors influencing soil quality and stability, which determine erosion dynamics and their impact on environmental recovery. These factors are categorized into five interrelated groups: soil properties, microbial communities, contaminants, economic considerations, and non-technical factors.

Soil is a dynamic and multifaceted ecosystem composed of a wide range of biotic and abiotic components that collectively create a favorable environment for plant development and support a rich diversity of microorganisms [228]. These elements are fundamental to maintaining soil health and delivering vital ecosystem services, including nutrient cycling, climate regulation, and organic matter decomposition (Figure 8) [229]. However, prolonged and intensive agricultural practices have resulted in severe soil degradation, manifested as nutrient depletion, compaction, erosion, and significant declines in microbial diversity [30]. Consequently, the soil’s capacity to sustain productive and resilient agricultural systems has been seriously impaired, with direct repercussions on both crop yield and quality [230].

To address these challenges, it is essential to implement soil management strategies that integrate biological inputs. These bio-inoculants contribute to the restoration and long-term preservation of soil health by enhancing nutrient availability and supporting sustainable agricultural practices, thereby reinforcing global food security.

Notably, recent long-term field trials and meta-analytical studies have confirmed the efficacy and scalability of microbial-based approaches to soil restoration [230]. For example, microbial consortia have demonstrated superior performances over single-strain inoculants in improving plant biomass and enhancing soil physicochemical properties across diverse agroecological contexts. Furthermore, meta-analyses have shown that bio-inoculants consistently increase soil organic carbon content, microbial biomass, and enzymatic activity over multiple cropping cycles [47].

A large-scale meta-analysis encompassing 335 studies revealed that microbial inoculants significantly enhanced soil microbial biomass, particularly under integrated fertilization regimes and in low-stress environments. Moreover, native inoculants tended to elicit stronger and more consistent effects compared to commercial or non-native strains [49]. Another study reported that inoculation with microbial consortia improved plant growth by 48% and pollutant remediation efficiency by 80%, underscoring the superior functional performance of diverse microbial communities under field conditions [231].

Collectively, these findings emphasize the importance of long-term, large-scale evaluations of microbial inoculants and the need for site-specific microbial formulations adapted to local soil types and climatic conditions. Such tailored solutions are essential to ensure the successful scaling of sustainable practices in real-world agricultural systems.

Plant growth and productivity are strongly limited by a variety of abiotic and biotic stressors [232,233]. Abiotic factors such as drought, heavy metal contamination, salinity, temperature extremes, and nutrient deficiencies induce profound physiological and morphological alterations that impair crop performance [234,235]. Concurrently, conventional intensive agricultural practices have amplified these challenges through the excessive application of chemical fertilizers, pesticides, and herbicides, leading to the depletion of beneficial soil microorganisms and disruption of microbial community structure [232].

**Figure 9:**
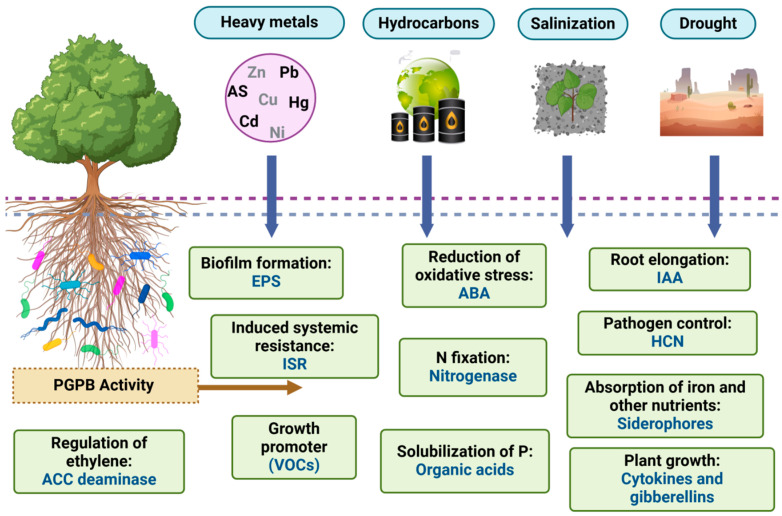
Primary adaptive responses promoted by PGPB to mitigate stress factors such as heavy metals, hydrocarbons, salinization, and drought, acting as microbial strategies to alleviate biotic and abiotic stress in plants. These microbial interactions contribute to the regulation of plant growth, enhancement of stress tolerance, and restoration of soil functionality, ultimately supporting plant resilience and sustaining agricultural productivity under environmental stress conditions.

The decline in microbial diversity, reduction in crop productivity, loss of soil fertility, and increase in interspecific nutrient competition are interrelated consequences of these stressors. Often, they act synergistically, exacerbating the severity of their individual effects and accelerating soil degradation [236]. In response to these adverse conditions, plants activate rapid defense mechanisms, such as the biosynthesis of PGRs and the accumulation of protective secondary metabolites, including phenolic acids and flavonoids [44]. Although these responses enhance plant resilience to environmental stress, they often come at the cost of reduced growth and productivity—illustrating the trade-off between survival and optimal yield (Figure 9).

### 4.1. Nutrient Dynamics in the Restoration of Degraded Soils

PSB not only improve soil fertility but also play a pivotal role in the rehabilitation of degraded soils [133]. These bacteria convert insoluble P compounds into bioavailable forms, thereby enhancing nutrient accessibility for plants. This process contributes to improved soil structure, enhanced aggregate stability, and stimulated root development, ultimately reducing erosion and increasing the soil’s water retention capacity [237]. In addition, PSB support the establishment of cover crops, which reduce soil exposure and further prevent erosion in degraded environments [130]. PSB also participate actively in the phosphorus cycle, ensuring sustained nutrient availability in ecosystems where the nutrient balance is frequently disrupted, a critical component in effective soil restoration [238]. Beyond their direct interactions with plants, PSB stimulate the proliferation of other beneficial microbial communities, thereby enhancing the decomposition of organic matter and nutrient recycling processes, leading to improved overall soil health [239].

Diazotrophic bacteria are equally essential in the restoration of degraded soils due to their unique ability to fix atmospheric nitrogen (N_2_) and convert it into plant-assimilable forms. This BNF is crucial for revitalizing soils depleted by intensive cultivation and nutrient loss. The fixed nitrogen not only supports the growth and development of the host plants but also enriches the surrounding soil, making nitrogen available to neighboring non-nitrogen-fixing species [240].

In addition to nitrogen fixation, diazotrophs contribute to the improvement of the soil structure through multiple mechanisms. They promote root development via the biosynthesis of PGRs, including auxins, cytokinins, and gibberellins [241]. Furthermore, these bacteria exude high-molecular-weight substances such as polysaccharides, EPSs, proteins, glycoproteins, and lipids that play crucial roles in the formation and stabilization of soil aggregates. EPSs typically contain sugars such as glucose, galactose, mannose, rhamnose, and uronic acids. These compounds form biofilms that promote bacterial adhesion to surfaces and facilitate microbial aggregation. The resulting biofilm matrix enhances the structural integrity of soil aggregates, improving both porosity and water-holding capacity [242].

Finally, the colonization and activity of diazotrophic bacteria support microbial biodiversity in the soil, thereby reinforcing ecological stability and resilience against environmental disturbances [243]. Altogether, these bacterial groups are critical in soil restoration strategies, contributing to fertility recovery, improved soil structure, and the sustained growth of healthy and productive vegetation.

### 4.2. Biological Strategies for Mitigating Salt-Induced Stress in Degraded Soils

Soil salinization is a multifaceted phenomenon resulting from processes such as nitrification, denitrification, organic matter decomposition, and excessive irrigation. These processes lead to the accumulation of soluble salts at the soil surface, including sodium sulfate, sodium chloride, magnesium sulfate, and magnesium chloride [244,245,246]. Elevated salt concentrations disrupt plant biochemical and physiological functions, particularly by impairing photosynthetic efficiency and cellular homeostasis [247].

The physical and chemical remediation of saline soil is often slow, economically unfeasible, and unsustainable. Furthermore, these methods can degrade soil structure, particularly in soil already burdened by high salinity [248]. As a sustainable alternative, halotolerant PGPB, such as *Bacillus halotolerans*, *Pseudomonas stutzeri*, and *Azospirillum brasilense*, have demonstrated efficacies in mitigating salt-induced stress in various plant species. For instance, *B*. *halotolerans* KKD1, isolated from saline soil, has been shown to enhance wheat growth under salt stress through the production of bioactive compounds and the modulation of stress-responsive gene expression [249]. Likewise, *P*. *stutzeri* MJL19, isolated from the rhizosphere of halophytic plants, significantly improves soybean germination and growth under saline conditions and exhibits enhanced biofilm formation under salt stress [250]. *A*. *brasilense*, particularly strain Sp245, has been widely studied for its ability to regulate the expression of genes related to salt stress tolerance, abscisic acid signaling, and nutrient transport in rice seedlings, thereby enhancing plant growth in saline environments [251]. These findings underscore the biotechnological potential of halotolerant PGPB as effective and sustainable tools for rehabilitating salt-affected soil.

The mechanisms by which PGPB alleviate salt stress include the accumulation of compatible osmolytes, elevated proline synthesis, selective uptake of K^+^ ions, reduction in electrolyte leakage, enhanced nutrient acquisition, nitrogen fixation, and the solubilization of phosphorus and other essential nutrients. Furthermore, these bacteria produce plant growth regulators that strengthen cellular defense pathways, siderophores that improve Fe availability, and EPSs that function as biocontrol agents and osmoprotectants, thereby improving plant tolerance to salinity. The expression of ACC deaminase further reduces stress by decreasing ethylene levels, which typically inhibit root elongation under stress conditions [37].

An additional key mechanism involves the regulation of aquaporin membrane proteins whose expression is induced under water-deficit conditions. Aquaporins facilitate water uptake and improve photosynthetic efficiency, thereby mitigating the adverse physiological impacts of salinity [252].

P uptake and translocation are highly sensitive to salinity, as phosphate often precipitates into insoluble forms under saline conditions, rendering it inaccessible to plants [253]. PGPB play a crucial role in mitigating this challenge through the solubilization of inorganic P, thereby increasing its availability and supporting plant growth in salt-affected soils [254]. This microbial intervention not only alleviates the negative effects of salinity but also promotes plant vigor and productivity under stressful conditions [255].

### 4.3. Bioremediation of Heavy Metal-Contaminated Soils Using PGPB

Industrialization, anthropogenic activities, and the excessive application of chemical fertilizers have contributed substantially to the accumulation of heavy metals (HMs) in soils, adversely affecting plant-associated microbial communities and posing serious risks to human and animal health [256]. This contamination is not limited to terrestrial ecosystems; HMs can leach into groundwater and bioaccumulate in agriculturally important plant species, increasing exposure risks through the food chain [257]. These metals exhibit mutagenic properties, induce DNA damage, and are associated with carcinogenic effects in humans and animals [258]. Long-term exposure to elevated concentrations of HMs has been linked to a range of detrimental health outcomes in humans and wildlife, establishing HM contamination as a critical global environmental concern [259].

**Figure 10:**
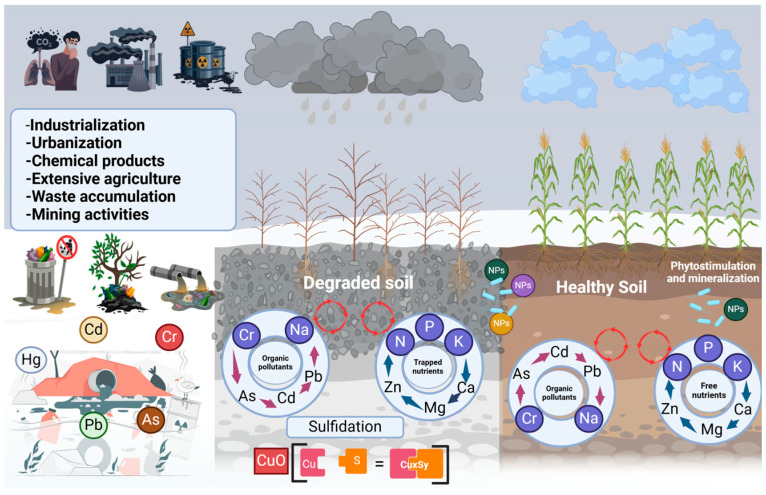
Soil degradation is driven by human activities such as industrialization, urbanization, chemical usage, extensive agriculture, waste accumulation, and mining, leading to the buildup of heavy metals (Hg, Cd, Cr, Pb, and As) and the depletion of essential nutrients (N, P, and K). In healthy soil, phytoremediation is enhanced by stimulating biological processes such as phytomineralization, which facilitate contaminant detoxification and nutrient recovery. Additionally, the use of nanoparticles (NPs) and sulfidation processes aid in stabilizing toxic elements.

Heavy metals are typically defined as elements with densities greater than 5 g/cm^3^ [260]. The most common HMs found in soil include manganese (Mn), copper (Cu), cadmium (Cd), lead (Pb), chromium (Cr), aluminum (Al), and zinc (Zn). Of the 53 identified heavy metals, 17 are biologically functional and essential for various physiological and ecological processes [257]. Some, including copper (Cu), iron (Fe), molybdenum (Mo), manganese (Mn), and boron (B), serve as micronutrients [261], while others, such as selenium (Se), arsenic (As), chromium (Cr), nickel (Ni), vanadium (V), and zinc (Zn), are considered trace elements [262]. In contrast, metals such as silver (Ag), mercury (Hg), lead (Pb), cadmium (Cd), and uranium (U) lack biological functions and are highly toxic to plants and soil microorganisms [263] (Figure 10).

Plant-associated microorganisms play a pivotal role in mitigating HM-induced stress by producing a range of beneficial metabolites, including PGRs, as well as ACC deaminase and siderophores [256].

PGPB exhibit multiple resistance strategies that enable them to survive and function in HM-contaminated soil. These mechanisms include metal exclusion via cell wall permeability barriers, active efflux transporters, extracellular and intracellular sequestration, modification of intracellular targets, enzymatic detoxification, and passive diffusion control. These responses are mediated by genes located on chromosomes or plasmids. Structural components, such as EPSs, lipopolysaccharides, the outer membrane, and the capsule, collectively reduce metal uptake by forming barriers or sequestering ions at the cell surface. Additionally, the genetic regulation of membrane channel proteins enables selective permeability, preventing toxic metal accumulation in the cytoplasm [264].

Despite the inherent toxicity of HMs to bacterial viability and metabolic activity, certain microbial populations have evolved adaptive strategies that allow them to tolerate and function under HM stress. These adaptations often result in shifts in microbial com-munity composition in both bulk soil and rhizosphere environments, with HM resistance frequently encoded on mobile genetic elements such as plasmids [257,258]. In addition to metal tolerance, these microorganisms retain essential ecological functions—including N fixation, siderophore production, P solubilization, and micronutrient mobilization—that collectively support phytoremediation and microbially assisted restoration of contaminated soils [265].

**Figure 11:**
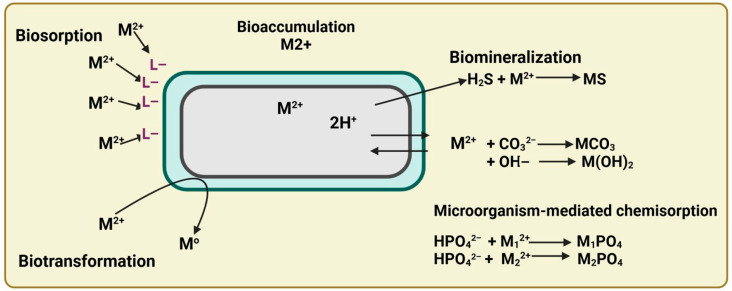
Mechanisms of interaction between HMs and microorganisms. Microorganisms engage with HMs through five primary pathways that facilitate their transformation, immobilization, or accumulation: biosorption (binding of metals to cell surface components), bioaccumulation (active transport and intracellular storage), biomineralization (conversion into mineral compounds such as sulfides or carbonates), biotransformation (modification of oxidation states, such as reduction to less toxic forms), and microbially mediated chemo adsorption (formation of complexes with phosphates, leading to immobilization).

PGPB further enhance plant resilience by modulating PGRs signaling pathways. They mitigate HM-induced oxidative stress by lowering ethylene accumulation through ACC deaminase activity and by regulating AUXs signaling, which promotes root elongation and nutrient acquisition [266]. The application of inoculants composed of HM-resistant PGPB, particularly when combined with phytoremediation strategies, offers an eco-compatible and sustainable solution for rehabilitating polluted soils (Figure 11) [100].

The synergistic interaction between plants and PGPB constitutes a robust biotechnological platform for the remediation of HM-contaminated environments. These bacteria not only attenuate metal toxicity through direct and indirect mechanisms but also restore soil functionality and enhance plant vigor, thereby supporting long-term productivity. Initially, PGPB research focused primarily on plant growth promotion and phytopathogen control. However, their relevance in environmental biotechnology has expanded significantly due to their capacity to degrade and mineralize a broad spectrum of organic pollutants in association with plants [258]. This multifunctionality underscores the growing potential of PGPB in integrated bioremediation systems.

Several native strains capable of degrading organic contaminants have been isolated from diverse ecological niches [267,268,269]. The genetic determinants underpinning these catabolic processes—including genes encoding oxygenases, hydrolases, and other degradative enzymes—have been extensively characterized, paving the way for targeted strain selection and metabolic engineering to enhance remediation efficiency [270].

## 5. Perspectives on Innovative Strategies for Soil Restoration Using PGPB in Overcultivated Lands

According to the FAO report, “The State of the World’s Land and Water Resources for Food and Agriculture”, the biophysical condition of approximately 5670 million hectares of land is declining globally. Of this total, 1660 million hectares (29%) have been degraded primarily due to human activities, while 4026 million hectares are undergoing degradation because of natural or anthropogenic pressures. Roughly half of these deteriorated lands are in poor condition, making them highly susceptible to further degradation. Additionally, 656 million hectares (12%) are under moderate pressure, posing future risks. Overall, 41% of global land degradation is directly attributed to unsustainable human activity [271].

This scenario highlights the urgent need for innovative and effective strategies to combat soil degradation, particularly due to its strong correlation with agricultural intensification and the escalating demand for food production. As the expansion of arable land becomes increasingly constrained, the restoration of degraded soils is essential to sustain crop yields and meet the nutritional needs of the growing global population [272].

At the 2023 United Nations Sustainable Development Goals (SDG) Summit, critical goals associated with soil and resource management—Zero Hunger (SDG 2), Climate Action (SDG 13), and Life on Land (SDG 15)—were emphasized. These goals form the foundation for achieving global food security and sustainable economic development [273]. In this context, the conservation of PGPB biodiversity has emerged as a key component in sustainable agricultural strategies, especially for rehabilitating degraded soils [274,275].

The implementation of microbial technologies in agroecosystems can substantially improve global food production, addressing one of the most persistent barriers to sustainable development: hunger [276]. In this regard, the use of PGPB offers an environmentally sound solution. Numerous studies have demonstrated that these microorganisms improve soil structure, promote rhizosphere regeneration, and enhance plant productivity in degraded soils, thereby supporting the transition to more sustainable agricultural systems [277].

Microbial inoculants possess significant potentials to restore soil functionality and sustain productivity in diverse agroecological contexts. However, while promising results have been observed under controlled conditions, field-level applications in degraded soil often yield variable outcomes. To address this, recent efforts have focused on the formulation of functionally synergistic PGPB consortia tailored to real-world conditions [278].

An emerging strategy involves combining PGPB consortia with compostable organic residues to improve their efficacy. For instance, a field study by Menguala et al. [279] demonstrated that inoculating *Lavandula dentata* L. seedlings with a rhizobacterial consortium and sugar beet residues significantly increased shoot and root biomass, as well as foliar nutrient content (NPK). Additionally, rhizospheric P and N availability increased by 29% and 46%, respectively, compared with the controls. The integration of selected rhizobacteria with organic substrates has been identified as a critical factor in optimizing microbial activity and enhancing the biotechnological potential of these formulations for the revegetation of degraded soils, particularly in semi-arid regions.

Similarly, the co-application of PGPB and biochar (BC) offers multiple advantages. BC enhances soil physicochemical properties, acts as a carrier matrix for bacterial inoculants, and provides a protective niche due to its porous structure. It also serves as a carbon source, facilitating microbial proliferation [217,280]. Studies have shown that PGPB–BC mixtures improve organic matter content, water-holding capacity, and microbial enzymatic activity especially those associated with stress mitigation, such as ACC deaminase production [281].

This strategy has been successfully applied in the remediation of soils contaminated with heavy metals [282,283] and hydrocarbons [284,285] as well as those affected by salinity [286,287,288]. Nonetheless, further long-term, field-based research is needed to confirm the consistency and scalability of these outcomes under diverse environmental and soil conditions.

Natural revegetation in soils degraded by long-term cultivation, particularly under rainfed conditions, is often hampered by water scarcity and poor soil structure. These limitations restrict plant establishment and slow ecosystem recovery [289]. Other factors, such as soil texture, nutrient status, and site-specific constraints, also influence the success of revegetation [290]. Consequently, restoration efforts have increasingly incorporated native plant species, which are better adapted to local conditions and more likely to establish successfully [188]. For example, Calle et al. [291] propose the use of native grasses and shrubs for reforesting arid and semi-arid lands in a strategy that has shown positive results in multiple regions [292,293].

The application of PGPB through seed coating or direct soil inoculation has proven to be a valuable strategy in these revegetation programs [51,294]. However, the success of such approaches depends on the development of inoculants capable of functioning under local environmental conditions, thereby optimizing nutrient uptake, water balance, and root development in restored plant communities [295].

Importantly, plants and their associated microbiomes often share adaptations to the same environmental niches. This ecological compatibility suggests that native microbial strains may be more effective in enhancing plant growth and resilience in degraded soils. In addition to promoting nutrient acquisition, native PGPB strains support biodiversity conservation and reduce the ecological risks associated with the introduction of non-native microbial species. As discussed previously, free-living bacteria can efficiently colonize the rhizosphere, stimulate root development, and promote plant fitness. Moreover, PGPB are known to synthesize vitamins and other co-factors essential for metabolic and physiological processes that underlie plant–microbe symbiosis [292].

## 6. Conclusions

Ensuring adequate agricultural production to sustain the global population amid accelerating soil degradation, environmental pollution, and increasing water scarcity remains one of the most pressing challenges of the 21st century. Simultaneously, the growing consumer demand for organic products reflects heightened concerns over the long-term impacts of agrochemical overuse, which has been shown to compromise soil integrity, reduce crop yields, and exacerbate ecological degradation.

In response, substantial research efforts have been directed toward the development of sustainable alternatives to synthetic agricultural inputs. Among these, PGPB have emerged as a promising biotechnological tool, offering immediate and measurable benefits for soil health, nutrient cycling, and plant development. Nonetheless, the field-scale application of PGPB in degraded soil remains inconsistent, primarily due to environmental variability and microbial survival constraints, underscoring the need for optimized inoculant formulations and delivery systems.

Recent findings highlight that the combination of PGPB with organic amendments, such as compostable residues and biochar, can significantly enhance microbial efficacy. These integrative strategies improve soil structure, increase nutrient bioavailability, and create favorable microhabitats for microbial colonization and persistence. Furthermore, the incorporation of microbial inoculants into revegetation efforts using native plant species has proven particularly effective for restoring ecosystem functionality in arid and semi-arid regions.

The synergistic use of PGPB with complementary soil restoration practices offers a scalable, eco-sustainable solution for rehabilitating degraded soil. Prioritizing continued research and long-term implementation of these integrated strategies is essential to mitigate soil degradation and ensure the resilience of global food systems in the face of climate change.

Emerging evidence supports the adoption of multi-faceted soil restoration approaches that enhance soil microbial diversity and ecosystem services. While the coming years will be decisive, the scientific community is well-positioned to advance microbial innovations that underpin sustainable agriculture and environmental restoration on a global scale.

## Data Availability

The data supporting this review will be made available by the authors on request.

## Abbreviations

The following abbreviations are used in this manuscript:

PGPB	Plant Growth-Promoting Bacteria
PGRs	Plant Growth regulators
PSB	Phosphate Solubilizing Bacteria
QS	Quorum Sensing
HM	Heavy Metals
